# Tomato spotted wilt virus in tomato from Croatia, Montenegro and Slovenia: genetic diversity and evolution

**DOI:** 10.3389/fmicb.2025.1618327

**Published:** 2025-07-28

**Authors:** Dijana Škorić, Jelena Zindović, Dorotea Grbin, Patrik Pul, Vladan Božović, Paolo Margaria, Nataša Mehle, Anja Pecman, Zala Kogej Zwitter, Denis Kutnjak, Ana Vučurović

**Affiliations:** 1 Department of Biology, Faculty of Science, University of Zagreb, Zagreb, Croatia; 2 Department of Plant Protection, Biotechnical Faculty, University of Montenegro, Podgorica, Montenegro; 3 Faculty of Food Technology, Food Safety and Ecology, University of Donja Gorica, Podgorica, Montenegro; 4 Plant Virus Department, Leibniz Institute DSMZ GmbH, Braunschweig, Germany; 5 Department of Biotechnology and Systems Biology, National Institute of Biology, Ljubljana, Slovenia; 6 School of Viticulture and Enology, University of Nova Gorica, Nova Gorica, Slovenia; 7 Jožef Stefan International Postgraduate School, Ljubljana, Slovenia

**Keywords:** tomato, HTS, phylogeny, TSWV, plant virus

## Abstract

Tomato spotted wilt orthotospovirus (TSWV) is a major plant pathogen causing significant economic losses in tomato production worldwide. Understanding its genetic diversity and evolutionary mechanisms is crucial for effective disease management. This study analyzed TSWV isolates from symptomatic tomato plants collected across Croatia, Montenegro and Slovenia between 2020 and 2024. High-throughput sequencing (HTS) was employed to obtain whole-genome sequences, followed by phylogenetic analyses to assess genetic variability and relationships among isolates from these three countries and other isolates of worldwide geographic origin. Phylogenetic analyses placed all studied isolates within the L1-M3-S3 genotype, commonly associated with solanaceous crops in Europe. While Croatian and Slovenian isolates exhibited high genetic similarity, Montenegrin isolates clustered in a distinct subgroup, showing closer relationships to Asian and Mediterranean accessions. Despite the severe disease symptoms observed, no substitutions in the NSm protein associated with resistance-breaking (RB) phenotypes were detected. These findings suggest that additional virome components, environmental factors or so far unknown mechanism(s) may contribute to infection and disease severity in tomato and strongly support the need of continuous surveillance of TSWV genetic diversity in order to inform breeding programs and develop sustainable management strategies to mitigate future outbreaks.

## Introduction

Tomato spotted wilt orthotospovirus (TSWV) is one of the most damaging re-emerging plant viruses. It is globally distributed, easily transmitted and has a very wide host range. These characteristics of TSWV, together with the severe disease symptoms in infected plants, manifested also on fruits, contribute to high yield losses in crop production worldwide amounting to more than one billion US dollars annually (Pappu et al., 2009; EFSA, 2012; Qi et al., 2021). TSWV (species *Orthotospovirus tomatomaculae*) is the most notorious member of the genus *Orthotospovirus* in the family *Tospoviridae* (EPPO, 2025).^1^ This family was formerly classified in the order *Bunyavirales* (Adkins et al., 2022; Zerbini et al., 2023) and has been only recently promoted to the class *Bunyaviricetes* (Kuhn et al., 2024) to better reflect the nature of this growing and diverse group of viruses. A study by Butković et al. (2021) delineated four distinct phylogroups within the genus *Orthotospovirus* based on the complete genome sequences of selected member species. These phylogroups contained viruses of diverse geographic origin, contradicting the previous study by Pappu et al. (2009) which had classified orthotospoviruses into two geographic groups: Asian and American. Based on the earlier work, TSWV was included in the American phylogroup (Pappu et al., 2009) while, the revised phylogeography proposes TSWV as the representative species of a phylogroup named A suggesting mainly an Asian origin (Butković et al., 2021).

TSWV is an enveloped virus with a segmented tripartite RNA genome (Pappu, 2008). The L segment (8.9 kb) is a negative-sense RNA containing a single open reading frame (ORF) in the viral genome complementary RNA strand (vcRNA) encoding the RNA-dependent RNA polymerase (RdRp), (Pappu, 2008; Tsompana and Moyer, 2008). The M segment (4.8 kb) is an ambisense RNA encoding the non-structural protein NSm in the viral genomic RNA (vRNA) strand, which functions as a viral movement protein. In the vcRNA strand, the M segment encodes a membrane glycoprotein precursor which is cleaved post-translation into Gn and Gc proteins, which are essential for assembling new virus particles and acquisition by the thrips vector (Pappu, 2008; Tsompana and Moyer, 2008). The S segment (2.9 kb) encodes the non-structural protein NSs in the vRNA strand, acting as a suppressor of plant RNA silencing defense responses. In the vcRNA strand, it encodes the nucleocapsid protein N stabilizing viral ribonucleoprotein complexes within the virus particle and during intercellular transport (Kulshrestha et al., 2013; Turina et al., 2016).

To date, more than 1,500 plant species have been identified as hosts of TSWV, including monocots and dicots, agronomically important crops and ornamentals, as well as weeds with a role in disease epidemiology (Oliver and Whitfield, 2016; EPPO, 2025; Qi et al., 2021; Fidan et al., 2024). Due to the severe economic damage it causes and its global distribution, TSWV has been included in the A2 list of the European and Mediterranean Plant Protection Organization (EPPO) as a quarantine pathogen recommended for regulation in some countries and geographical regions (EPPO, 2025). The most affected crops in the *Solanaceae* family are tomato (*Solanum lycopersicum*) and pepper (*Capsicum annuum*) (Pappu et al., 2009). The “spotted wilt of tomato” disease caused by TSWV was first reported in Australia in 1915 and later determined to be of viral origin (Samuel et al., 1930). Regardless of the name, the symptoms of this disease can vary considerably and depend on numerous factors, such as the host species, cultivar, its genetic profile and developmental stage, environmental conditions, strain, and possibly co-infections with other viruses (García-Cano et al., 2006; Oliver and Whitfield, 2016; EPPO, 2025). While TSWV symptomatic infection can include both local and systemic symptoms varying in severity, asymptomatic infections are also quite common, especially in weeds, which can serve as the virus reservoir hosts for subsequent infections of crops (EPPO, 2025; Fidan et al., 2024). Tomato plants may show various leaf symptoms including mosaic, mottling, chlorosis, bronzing, ring-shaped chlorotic spots and necrotic lesions. Fruits may develop necrotic or chlorotic spots and concentric rings. In addition, the whole plant may show severe stunting, leaf deformation, stem necrosis and wilting (de Ronde et al., 2019; Batuman et al., 2020). In nature, TSWV is primarily transmitted horizontally by thrips, insect vectors of the order *Thysanoptera*. At least ten thrips species (Caruso et al., 2024) have been identified as vectors, the most important being the western flower thrips *Frankliniella occidentalis* (Rotenberg et al., 2015). Infections of thrips with TSWV are persistent and propagative, which together with the high reproductive potential of the vector, its short generation time, exceptional mobility, polyphagia and resistance to insecticides, contribute to efficient virus transmission (Rotenberg et al., 2015; Gupta et al., 2018). TSWV can also be transmitted by vegetative propagation of plant hosts and there is limited evidence of transmission via seed and pollen (EPPO, 2025; Wang et al., 2022), however, there is no such evidence in tomato.

In addition to controlling the thrips vectors and reservoir hosts, a key component of the most effective strategy currently used to prevent disease caused by TSWV infections in tomato is the planting of resistant cultivars containing the *Sw-5b* gene (EFSA, 2012; Batuman et al., 2020). This gene has been introduced into various commercial cultivars by introgression from a wild tomato species, *Solanum peruvianum*, and confers resistance to a wide range of TSWV strains (Stevens et al., 1991). The widespread breeding and employment of resistant cultivars has inevitably led to the emergence of TSWV isolates with a resistance-breaking (RB) phenotype through natural selection (Peiró et al., 2014; de Oliveira et al., 2018) Initially, two substitutions in the NSm protein associated with the RB phenotype were identified by molecular characterization and experimental mutagenesis, namely the amino acid substitutions C118Y and T120N (López et al., 2011; Peiró et al., 2014; Huang et al., 2022). Recently, two new substitutions associated with the RB phenotype in tomato have been reported, D122G in Hungary (Almási et al., 2023), also described from the United States (Lahre et al., 2023), and C118F, restricted so far to Mexico (Rodríguez-Negrete et al., 2023). It is important to note that the appearance of symptoms on tomato plants with the *Sw-5b* resistance gene does not always indicate the presence of the described TSWV RB isolates. Other factors may also influence resistance, such as co-infection with other viruses as the case tomato chlorosis virus (ToCV) (García-Cano et al., 2006) and the emergence of more severe TSWV strains. Environmental conditions such as high temperatures also could affect the number of local lesions in resistant tomato varieties (Mitidieri et al., 2001). Therefore, it is necessary to study the intrinsic diversity of TSWV as it contributes to the epidemiological key features like disease symptom types and severity, transmission efficiency, and breaking of plant host resistance (Briese et al., 2013; Butković et al., 2021; Siampour et al., 2024).

In Croatia, TSWV was first described in tobacco in 1978 (Bužančić and Juretić, 1978). Over the years, several outbreaks have been reported in pepper (Škorić et al., 1997), tomato, lettuce and chrysanthemum (Ivić et al., 2016), with escalating problems in greenhouse vegetable production. The first report of TSWV in Slovenia, affecting tomato, pepper, chrysanthemum, calla lily, cyclamen and spatiphylum, dates back to 2000 (Mavrič and Ravnikar, 2001). At that time, *Artemisia vulgaris*, a weed growing outside the greenhouses, was also found to be infected. Since then, TSWV has been commonly detected in various plants across Slovenia, and the most recent comprehensive virome study on tomato and surrounding weeds revealed the presence of TSWV in both tomato and weeds (Rivarez et al., 2023). In Montenegro, the presence of TSWV was initially detected in 2007 in two imported *Dracaena* sp. plants. Subsequent surveys confirmed the occurrence of the virus in pepper and a range of ornamental plants including *Calceolaria* sp., *Primula* sp., *Petunia* sp., *Aquilegia* sp., *Gerbera* sp., and *Chrysanthemum* sp. (Zindovic et al., 2014).

In this study, samples of symptomatic tomatoes collected in Croatia, Slovenia and, to a limited extent, Montenegro, over a 5-year period (2020–2024) were screened for TSWV infection using various immunochemical and molecular methods. The whole genome or genomic segment sequences of TSWV isolates from infected tomato samples were obtained by high-throughput sequencing (HTS) of 18 sequencing libraries, and their molecular characteristics were analyzed, including the genetic variability, phylogeny, and the presence of substitutions associated with the RB phenotype in the NSm protein sequence. We conducted comprehensive phylogenetic analyses to elucidate the molecular relationships among TSWV isolates from these three neighboring countries. Our study aimed to compare these isolates with those from other regions worldwide, providing a broader context for understanding their genetic diversity. Additionally, we sought to identify potential pathways through which this significant plant pathogen might have entered the above-mentioned countries.

## Materials and methods

### Collection of tomato samples and preliminary screening

Tomato plants from protected growing facilities (greenhouses) and open fields showing virus-like symptoms were screened for viruses at different localities in all three countries over five growing seasons (2020–2024) (Supplementary Figure S1). In Slovenia, the inspections covered the areas with favorable conditions for tomato cultivation, including the continental and warmer localities of the Slovenian Adriatic coastline. Although Croatian samples were not as numerous as Slovenian ones, and all of them arose from greenhouse cultivations, they originated from two continental localities about 100 km apart and from one locality in Dalmatia (the central part of the eastern Adriatic coast), thus covering two main agro-ecological niches of tomato cultivation in this country. The Montenegrin samples were collected from greenhouses in the southernmost part of the Montenegrin territory on the eastern Adriatic coast (for details see Supplementary Table S1).

Besides symptomatology, immunochemical methods like immunostrips (ImmunoComb for CMV, INSV, TMV, TSWV, Agdia, United States) or double-antibody sandwich enzyme-linked immunosorbent assay (DAS-ELISA TSWV, Bioreba AG, Switzerland) were used for TSWV screening in Croatia and Montenegro, respectively. In Slovenia, tomato samples were collected during the official surveys for tomato brown rugose fruit virus (ToBRFV) from 2020 to 2024. Symptomatic samples in which ToBRFV was not confirmed were analyzed by HTS. In total, 18 libraries of pooled or single tomato samples showing necrosis or chlorosis on the leaves and/or fruits were selected for HTS either by using Illumina or Oxford Nanopore Technologies (ONT) platforms (for Croatian and Montenegrin samples the selection was based on positive TSWV screening results). HTS libraries originated from a total of 49 tomato samples: 40 from Slovenia, 7 from Croatia and 2 from Montenegro (Table 1; Supplementary Table S1).

**Table 1:** Overview of sequenced libraries and NCBI GenBank accession numbers of sequences of TSWV isolates from Slovenia, Croatia and Montenegro obtained in this study.

Library/isolate name	Library type	Country	Year of sampling	HTS platform	GenBank AN for S segment/N, NSs	GenBank AN for M segment/GnGc, NSm	GenBank AN for L segment/RdRp
52STT21S	Single	Croatia	2021	Illumina	PV445500	PV445499	PV445498
104DOT22S	Single	Croatia	2022	Illumina	OQ534349	OQ534350	OQ534351
105DOT22S	Single	Croatia	2022	Illumina	OQ507121	OQ507122	OQ507123
107DOT22S	Single	Croatia	2022	Illumina	OQ507124	OQ507125	OQ507126
108DOT22S	Single	Croatia	2022	Illumina	OQ507127	OQ507128	OQ507129
106DOT22S	Single	Croatia	2022	Illumina	OQ534340	OQ534341	OQ534342
71SET22S	Single	Croatia	2022	Illumina	OQ534343	OQ534344	OQ534345
98/23	Single	Montenegro	2023	Illumina	PP968743	PP968742	PP968741
100/23	Single	Montenegro	2023	Illumina	PP968746	PP968745	PP968744
D262/20	Single	Slovenia	2020	Illumina	PV092087, PV092069	PV092081, PV092071	na
D474/20	Single	Slovenia	2020	Illumina	PV092089, PV092070	PV092083, PV092072	na
D599/20	Single	Slovenia	2020	Illumina	PV092090, na	PV092085, PV092073	na
D_K3_21	Pooled	Slovenia	2021	Illumina	PV092091, PV092065	PV092084, PV092074	PV092062
D_K4_21	Pooled	Slovenia	2021	Illumina	PV092092, PV092066	PV092082, PV092075	PV092063
D_K5_21	Pooled	Slovenia	2021	Illumina	PV092093, PV092067	PV092086, PV092076	PV092064
D_P4_22	Pooled	Slovenia	2022	ONT	PV09294, na	na, PV092077	na
D_P10_22	Pooled	Slovenia	2022	ONT	PV09295, na	na, PV092078	na
D_P2_24	Pooled	Slovenia	2024	ONT	PV092096, PV092068	PV092080, PV092079	na

AN, accession number; na, no sequence was generated due to low read coverage; ONT, Oxford Nanopore Technologies.

### Sample preparation and HTS

For samples originating from Croatia, total nucleic acids were extracted from 500 mg of leaf, or fruit skin (Supplementary Table S1) tissue of each plant by CTAB-based method (Murray and Thompson, 1980) and DNase treated as instructed by the manufacturer (RQ1 RNase-Free DNase, Promega, United States). After the measurement of RNA concentration and purity (NanoDrop ND-1000, NanoDrop Technology, United States), resulting total RNAs were depleted of host rRNAs (RiboMinus Plant Kit for RNA-Seq, Thermo Fisher Scientific, United States), polyA tailed with *E. coli* Poly(A) Polymerase (New England Biolabs, United States) according to the manufacturers’ instructions and sent for downstream preparation steps and sequencing (Illumina total RNA-Seq, NovaSeq, 2 × 150 bp) as individual samples (Supplementary Table S1) to a commercial service (Genewiz, Azenta Life Sciences, United States). Raw sequence data were submitted to the National Center for Biotechnology Information (NCBI) SRA database under accession number PRJNA946736.

For samples from Montenegro, total RNAs were extracted by CTAB-based method from 100 mg of tissue according to a published protocol (Zindović et al., 2024) and sent to the Leibniz Institute - German Collection of Microorganisms and Cell Cultures (DSMZ) laboratories (Germany) where they were treated with DNase I (Ambion, United States) and thereafter used as input for double-strand cDNA synthesis (Maxima H Minus Reverse Transcriptase, Thermo Scientific, United States; NEBNext Ultra II Non-Directional RNA Second Strand Module, New England Biolabs, United States), including a ribosomal RNA-depletion treatment (QIAseq FastSelect–rRNA Plant kit, Qiagen, United States). The libraries were prepared by using an Illumina DNA Prep Kit (Illumina, United States) and sequenced on a NextSeq2000 (Illumina) instrument as paired-end reads (2 × 150 bp). Raw sequence data were submitted to the NCBI SRA database under accession number PRJNA1219120.

For samples originating from Slovenia, total RNAs were extracted from leaf and/or fruit skin material (approximately 200 mg) using RNeasy Plant mini kit (Qiagen, United States), following the manufacturer recommendations, with minor modifications: specifically, without using 2-mercaptoethanol, and performing the final RNA elution with two consecutive washes with 50 μL (total of 100 μL) of RNase-free water pre-warmed to 65°C. To assess the quality of the RNA extractions, RNA samples were tested with reverse transcription quantitative real-time PCR (RT-qPCR) using nad5-specific primers and a probe (Menzel et al., 2002). RNA extraction was considered successful if obtained quantification cycles (Cq) value for nad5 was equal to or less than 33, otherwise the RNA extraction was repeated. Extracted RNAs for samples from 2020 were sent to SeqMatic LLC (United States) for library preparation and sequencing, on MiSeq (Illumina) as paired-end reads (2 × 250 bp). Total RNA of samples from 2021 were pooled in three pools by mixing equal mass of RNAs originating from different samples to ensure a uniform concentration of each sample in the pool and sent for library preparation and sequencing on Illumina NovaSeq (Novogene, UK) as paired end reads (2 × 150 bp). For samples from 2022 and 2024 libraries were prepared and sequencing was performed in-house using MinION (ONT) device as pooled samples (in equal mass ratio of RNAs). Samples from 2022 were sequenced as two pools using MinKNOW software (version 22.03.5) flow cell FLO-MIN106 (ONT) and sequencing kit SQK-PCB109 (ONT), while samples from 2024 were sequenced as one pool (MinKnow 24.06.5, flow cell FLO-MIN114, kit SQK-PCB114-24, ONT). For more details on samples refer to Supplementary Table S1. Raw sequence data were submitted to the NCBI SRA database under accession number PRJNA1219120.

### Bioinformatic analyses of HTS datasets

HTS datasets were subjected to different analysis pipelines, depending on the sequencing method used and the laboratory of origin. For Croatian samples, reads were quality checked and trimmed by Cutadapt (Martin, 2011). Paired-end reads were then merged using FLASh. Taxonomic classification was performed using Kraken2 (Wood et al., 2019) against a custom viral database (version Jan 13, 2023). Reads were subsequently aligned to a reference viral genome using BWA-MEM (Li and Durbin, 2009), and variant calling and consensus sequence generation were performed using SAMtools and BCFtools (Li et al., 2009; Li, 2011). The obtained contigs were screened by BLASTn and BLASTp against a custom database for plant virus/viroid discovery. Taxonomic assignment of the reads was conducted in DIAMOND (v. 2.0.8)/BlastX (database v. July 16, 2022) (Buchfink et al., 2015) against a protein database and MEGAN6 (v. 6) (Huson et al., 2016).

For Montenegrin samples, in brief, the raw reads were imported into Geneious Prime software version 2023.1.1 (Dotmatics, United States), paired, trimmed, mapped against host sequences (chloroplast genome sequence accession PRJNA16427; mitochondrion genome sequence accession PRJNA413096; chromosomes and transcript sequences accession PRJNA66163), and the resulting un-mapped reads were *de novo* assembled using the in-house pipeline developed at DSMZ. The obtained contigs were screened against a custom virus reference database using BLASTn and BLASTp alignment for virus discovery and taxonomic assignment, and final genome sequences were assembled by manual curation and comparison with sequences available in NCBI GenBank database.

For Slovenian samples sequenced by Illumina platform, bioinformatic analyses were performed using the pipeline developed by Pecman et al. (2022) in CLC Genomic Workbench (Qiagen, United States) v. 20–23. To achieve potentially improved assemblies of viral genomes, we also performed *de novo* assembly for all samples using SPAdes version 3.6.1 (Bankevich et al., 2012). We used the paired-end read library input file and set k-mer lengths to 21, 33, 55, and 77 with the –careful option. Consensus sequences of detected TSWV genome segments or genes were reconstructed using *de novo* assembled contigs and/or iterative read mapping to the most similar reference sequences from NCBI GenBank. ONT sequenced libraries were analyzed using the bioinformatics pipeline of Pecman et al. (2022) with the only exception of sample from 2024, where base calling was performed using Dorado software v 0.7.2 (ONT) and all other steps were the same. Consensus sequences of detected TSWV genome segments or genes were reconstructed as described for the Illumina samples, except that read/contig mapping was performed using minimap2 implemented as the Long Read Support (beta) plugin of CLC GW v. 22–24 and default parameters. The 5x read depth coverage cutoff was applied when exporting final consensus sequences.

The number and average length of the sequencing reads for each library from this study are detailed in Supplementary Table S2. The number and average length of the sequencing reads generated for each library from this study are detailed in Supplementary Table S2. All TSWV genome sequences obtained in this study have been submitted to NCBI GenBank (Table 1).

### Phylogenetic analyses and pairwise sequence similarity comparisons

To analyze phylogenetic relationships among TSWV isolates from this study and other isolates globally recorded, the herein obtained TSWV sequences (Table 1; Supplementary Table S1) were used alongside sequences retrieved from GenBank for all three genomic segments of TSWV representative isolates (Supplementary Table S3). The sequences of TSWV genomic segments were analyzed separately for the RdRp gene (large segment, L), GcGn and NSm genes (medium segment, M), and N and NSs genes (small segment, S).

Sequences were aligned using Clustal Omega (version 1.2.4) available at the EMBL-EBI website^2^ (Madeira et al., 2022), with default parameters (input alignments used in the analyses are available in Supplementary Table S4). Maximum likelihood phylogenetic trees were constructed using IQ-TREE (online version 1.6.12) using five different alignments previously described (RdRp, GcGn, N and NSs). The best-fit substitution model was selected based on the Bayesian Information Criterion (BIC), and branch support was evaluated using 1,000 ultrafast bootstrap replicates and the SH-like approximate likelihood ratio test (aLRT) with 1,000 replicates. Finally, the phylogenetic trees were visualized, midpoint rooted and annotated using the Interactive Tree of Life (iTOL) platform (Letunic and Bork, 2024, version 7^3^).

To explore the relationships of TSWV isolates and detect potential reassortment signals, a phylogenetic network analysis was performed in SplitsTree 6.4.13 (Huson and Bryant, 2024). Concatenated alignment of RdRp, GcGn, NSm, N and NSs gene sequences from two Montenegrin, seven Croatian and three Slovenian TSWV isolates, along with 38 TSWV sequences from NCBI GenBank databases, was subjected to analysis (Supplementary Tables S1, S4). Default settings were used, implementing Neighbor-Net analysis. Bootstrapping was conducted with 1,000 replicates.

Average nucleotide sequence identities within and between different phylogroups in the phylogenetic trees were calculated using the p-distance model in Mega11 (Tamura et al., 2021). Nucleotide sequences of the complete TSWV genomes (all three segments) and genes datasets from Croatia (7 isolates), Montenegro (2) and Slovenia (3) were aligned by ClustalW and pairwise sequence identity scores were calculated in Mega11.

### Recombination analysis

To identify recombination patterns and potential recombination break points within the 12 TSWV isolates from this study for which all genes were obtained, we used the Recombination Detection Program version 4.101 (RDP4) (Martin et al., 2015). Aligned concatenated RdRp, GcGn, NSm, N and NSs gene sequences from two Montenegrin, eight Croatian and three Slovenian TSWV isolates, along with 38 sequences from NCBI GenBank databases, were subjected to analysis of potential recombination or reassortment events using RDP4 software with default parameters and nine different algorithms (RDP, GENECONV, Bootscan, Maxchi, Chimaera, SiScan, 3Seq, PhylPro, and LARD). Recombination events predicted by four or more methods with a probability value threshold of 0.01 were considered evidence of a putative recombination event.

## Results

### Tomato samples

Collected tomato plants showed typical virus-like symptoms including chlorosis, bronzing, leaf and stem necroses, stunted growth and wilting. Tomato fruits mostly exhibited severe symptoms beginning with chlorotic ringspots and uneven ripening, which progressed to severely necrotic rings and patches making fruits completely unmarketable (Supplementary Figure S1; Supplementary Table S1). As diverse tomato cultivars were included in the analyses (including different growth types and fruit shapes), as well as unknown cultivars, it was not possible to conduct comparative phenotypic analyses. Observed disease incidence varied between countries. In Montenegro, TSWV incidence in tomato crops was 7% in 2023. In Slovenia, TSWV was sporadically detected between 2020 and 2024 in both individual and pooled tomato samples. For example, in 2020, three out of four samples tested positive, while in subsequent years the virus was confirmed in a limited number of pooled samples, each comprising 6–7 plants. In Croatia, TSWV was detected in one out of 32 tested samples in 2021, and in six out of 51 tested samples in 2022. However, due to non-systematic sampling across diverse cultivars and locations, no reliable estimation of disease incidence could be made for Slovenia and Croatia.

A total of 18 HTS libraries which included 49 tomato samples from the three considered countries were analyzed, with the majority from Slovenia (82%), 14% from Croatia and 4% from Montenegro (Supplementary Table S1).

### Reconstructed TSWV partial and complete genomic sequences

Full-length genome sequences or complete sequences for all five ORFs were obtained for 12 TSWV isolates: seven from Croatia, two from Montenegro, and three from Slovenia. Partial genomic sequences, where one or more ORFs were missing, were obtained for additional six isolates from Slovenia (NSm and N sequences were obtained for all six isolates, GnGc sequences for four, NSs sequence for three, and the RdRp sequences were missing for all six). All sequences obtained by HTS in this study were deposited in the NCBI GenBank as individual sequences for each of the virus genomic segments or genes, their NCBI GenBank accession numbers are available in Table 1 and Supplementary Table S1.

### Pairwise sequence similarity analysis of Slovenian, Croatian and Montenegrin TSWV isolates

This analysis was performed for isolates for which complete sequences of all ORFs were reconstructed from HTS data. Accordingly, the analysis included pairwise comparisons of concatenated nucleotide sequences of all five genes of three Slovenian, seven Croatian and two Montenegrin TSWV isolates (Tables 1, 2). Overall, pairwise nucleotide identities among TSWV isolates from Croatia, Slovenia and Montenegro ranged from 97.4 to 100%. The analysis showed that Montenegrin isolates were more dissimilar to Croatian and Slovenian isolates (pairwise nucleotide sequence identities in range of 97.4–97.8%), than Croatian and Slovenian isolates among each other (98.5–99.7%). Croatian isolates were 99.6–100% identical among themselves, two Montenegrin were 99.6% identical, and three Slovenian isolates showed the biggest within-country diversity (98.6–99.3% pairwise identities).

**Table 2:** The pairwise nucleotide sequence identities (in %) of concatenated sequences of RdRp, GcGn, NSm, N, and NSs genes of Slovenian, Croatian and Montenegrin TSWV isolates obtained in this study.

	D-K4-21	52STT21S	D-K3-21	D-K5-21	71SET22S	105DOT22S	104DOT22S	107DOT22S	108DOT22S	106DOT22S	98/23	100/23
D-K4-21												
52STT21S	98.6											
D-K3-21	98.6	99.6										
D-K5-21	98.6	99.2	99.3									
71SET22S	98.6	99.8	99.7	99.2								
105DOT22S	98.6	99.7	99.6	99.2	99.9							
104DOT22S	98.6	99.6	99.6	99.2	99.9	99.9						
107DOT22S	98.5	99.6	99.6	99.2	99.9	99.9	100					
108DOT22S	98.5	99.6	99.6	99.2	99.9	99.9	100	99.9				
106DOT22S	98.5	99.6	99.6	99.2	99.9	99.9	100	100	99.9			
98/23	97.4	97.7	97.8	97.8	97.8	97.7	97.7	97.7	97.7	97.7		
100/23	97.4	97.8	97.8	97.8	97.8	97.8	97.8	97.7	97.7	97.8	99.6	

Self-comparisons are omitted.

Orange shading denotes pairwise nucleotide similarities between Slovenian TSWV isolates, green between Croatian isolates, and blue between Montenegrin isolates.

### Resistance-breaking amino acid motives analysis

**Figure 1:**
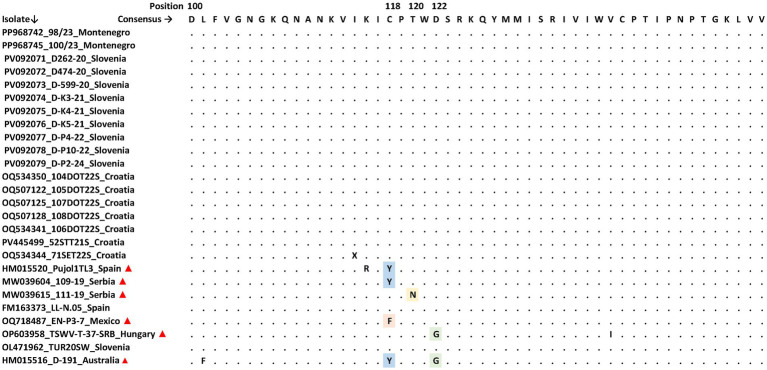
TSWV NSm amino acids alignment of 18 Slovenian, Croatian, and Montenegrin isolates from this study with TSWV-RB (▲) and other TSWV isolates from GenBank. Substitutions C118Y/F (cytosine to tyrosine or phenylalanine), T120N (threonine to asparagine) or D122G (aspartic acid to glycine) described for TSWV-RB strains are highlighted. Indicated after the accession number of each isolate are the isolate ID and the country of origin.

The positions of aa substitutions in the NSm protein sequence linked to the RB TSWV strains described so far (C118Y/F, T120N, and D122G) were taken into account for the aa sequence alignment including the aa region 100–150 of the NSm protein of 18 Slovenian, Croatian, and Montenegrin TSWV NSm sequences obtained in this study and a subset of RB TSWV and wild-type strains retrieved from GenBank (Figure 1). The previously described RB phenotype related substitutions did not occur in the isolates investigated in this study, neither new substitutions unique for isolates included here could be recorded in this NSm region.

### Phylogenetic relationships

**Figure 2:**
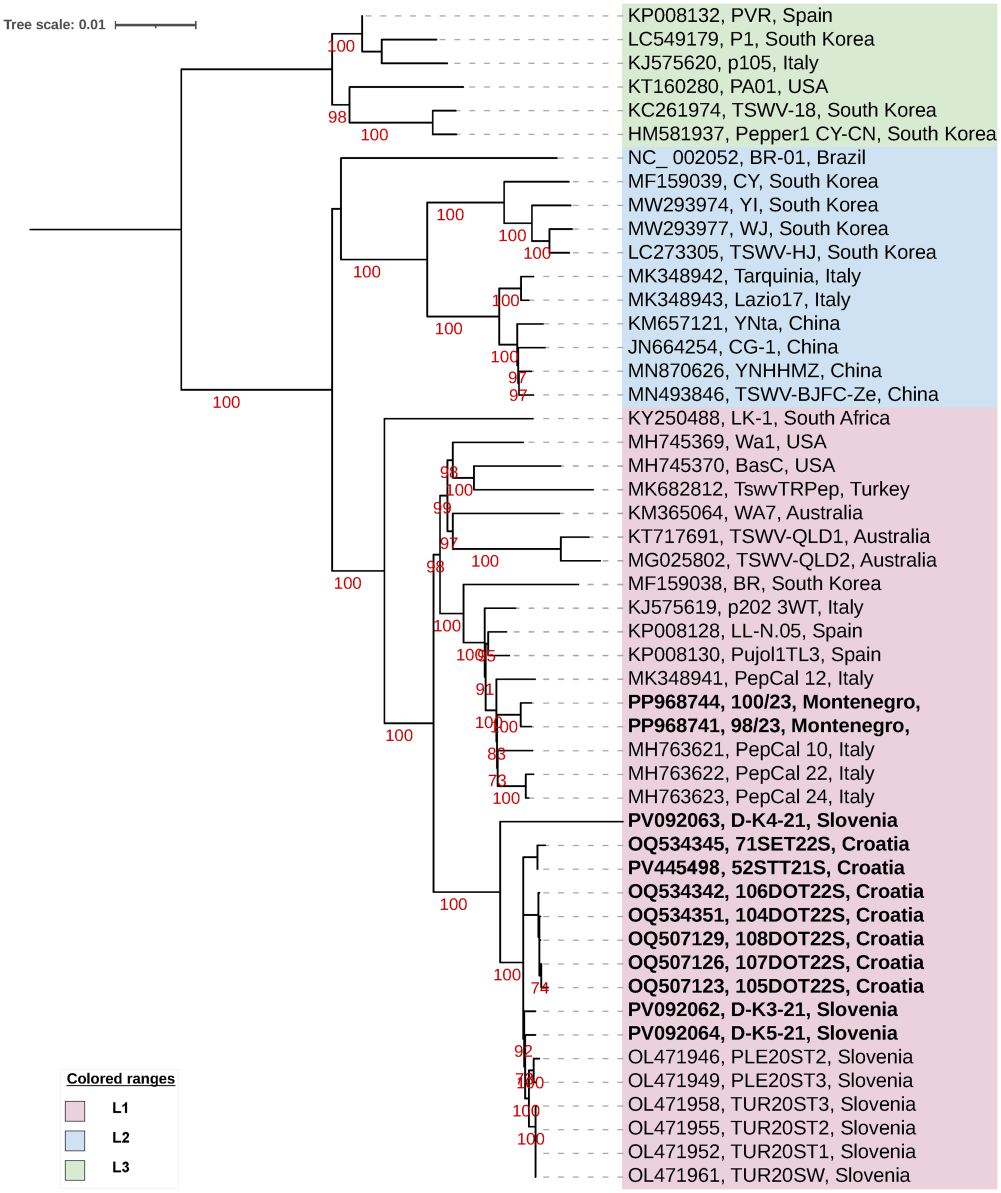
A maximum likelihood phylogenetic tree based on the alignment of the full-length RdRp gene sequences of 50 TSWV isolates. The phylogram was generated with IQ-TREE using the Best-fit model: TN + F + I + G4. The bootstrap analysis was performed with 1,000 replicates, and bootstrap values (>70%) are shown next to the relevant branches. The tree was midpoint rooted. The TSWV isolates generated in this study are in bold. Leaf labels consist of NCBI accession number of isolate RdRP sequence, the isolate ID and country of origin. The tree scale represents the number of substitutions per site.

**Figure 3:**
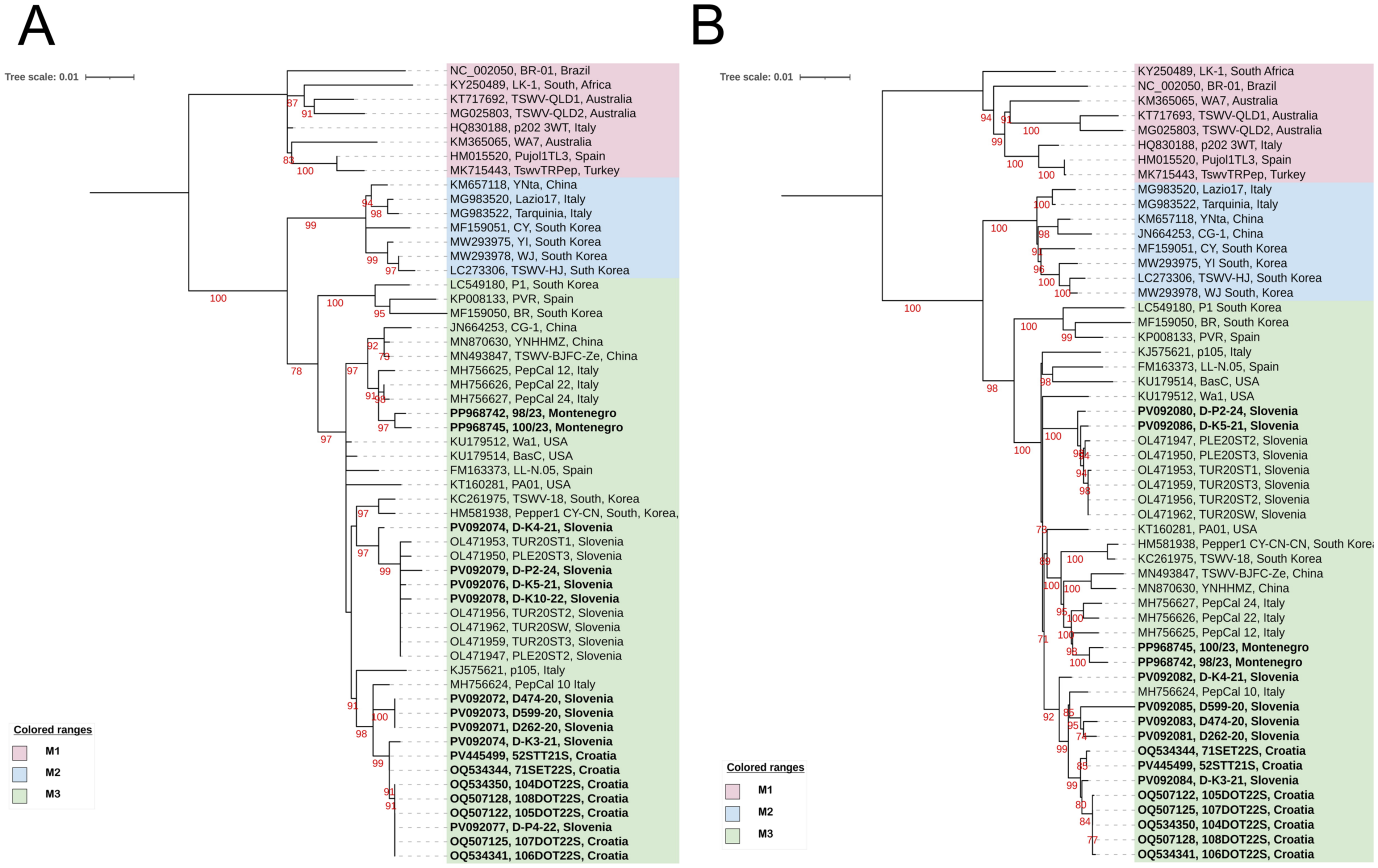
A maximum likelihood phylogenetic trees based on the alignment of the full-length: **(A)** NSm gene sequence of 56 TSWV isolates. The phylograms were generated with IQ-TREE using the Best-fit model: HKY + F + G4; and **(B)** GnGc gene sequences of 54 TSWV isolates. The phylogram was generated with IQ-TREE using the Best-fit model: TN + F + G4. The bootstrap analysis was performed with 1,000 replicates, and bootstrap values (>70%) are shown next to the relevant branches. The trees were midpoint rooted. The TSWV isolates generated in this study are in bold. Leaf labels consist of NCBI accession number of isolate NSm **(A)** or GnGc **(B)** sequence, the isolate ID and country of origin. The tree scale represents the number of substitutions per site.

**Figure 4:**
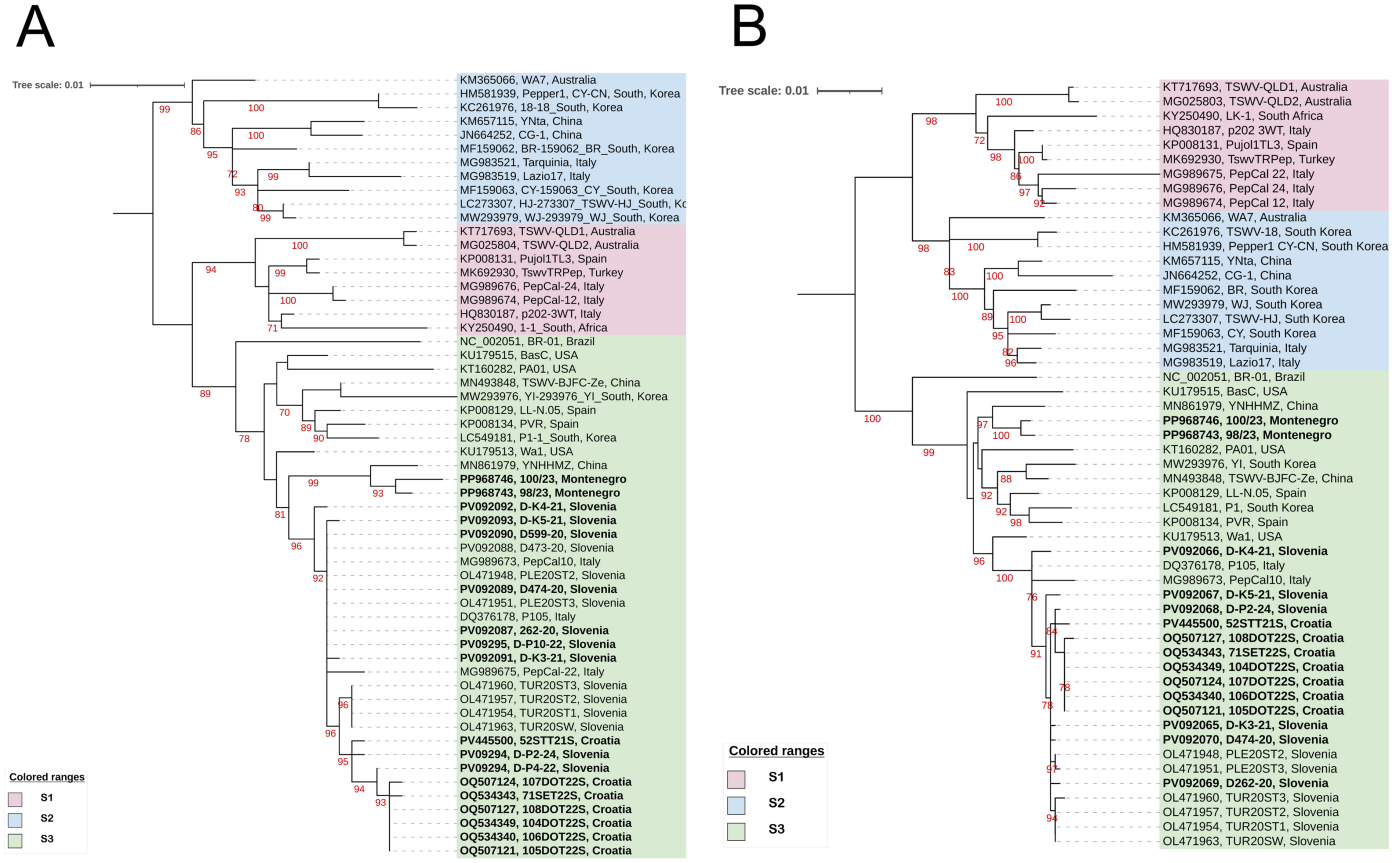
A maximum likelihood phylogenetic trees based on the alignment of the full-length: **(A)** N gene sequences of 57 TSWV isolates and **(B)** NSs gene sequence of 53 TSWV isolates. The phylograms were generated with IQ-TREE using the Best-fit model: HKY + F + G4. The bootstrap analysis was performed with 1,000 replicates, and bootstrap values (>70%) are shown next to the relevant branches. The trees were midpoint rooted. The TSWV isolates generated in this study are in bold. Leaf labels consist of NCBI accession number of isolate N **(A)** or NSs **(B)** sequence, the isolate ID and country of origin. The tree scale represents the number of substitutions per site.

Phylogenetic analyses (Figures 2–4) of genes from all three TSWV genomic segments showed clear differentiation of three clades with high statistical support of the groupings, as demonstrated from the high bootstrap values for the branches leading to those three groups. The clades were denoted as in Siampour et al. (2024) based on genomic segment letter and clade number (e.g., L1-M3-S3).

In the RdRp gene analysis (Figure 2) sequences from the three studied countries clustered together in clade L1 (Siampour et al., 2024), D-like in Fontana et al. (2020) wherein all Slovenian and Croatian sequences belong to the same subgroup while two Montenegrin sequences are more similar to Italian, Spanish and other globally sourced accessions in another subgroup of the same clade. The average nucleotide sequence identity in the RdRp gene among the three L clades ranges from 93.9 to 95.6% (Supplementary Table S5). The average nucleotide sequence identity within each clade ranges from 97.7 to 98.1% with the clade L1 exhibiting the highest degree of sequence conservation among the three clades (Supplementary Table S5).

Based on the alignment of the genes on the M and S segments (Figures 3, 4), the TSWV sequences from the three studied countries were not clustered within clades M1 and S1 (Siampour et al., 2024), respectively, as the L-segment gene, but within the clades M3 and S3, described as A-like clades in Fontana et al. (2020). Phylogenetic relationships among sequences studied here for the GcGn and NSm genes are represented with trees having slightly different topologies compared to each other. The GcGn tree (Figure 3B) for the clade M3 has four subgroups with major part of the Slovenian and Croatian isolates in one subgroup, and only two Slovenian isolates studied here in another. Montenegrin isolates are in a third, clearly distinct, subgroup clustered together with Italian and Asian isolates from non-tomato hosts (Supplementary Table S3). Within the clade, the average nucleotide sequence identity for GcGn gene is 98.0% and the average sequence identities among the three clades vary from 93.4 to 96.4% (Supplementary Table S5). For NSm (Figure 3A), phylogeny is slightly more complicated due to polytomy of the United States isolates and a Spain isolate in the clade M3 and geographically more diversified subgroups including all Croatian and five out of nine Slovenian isolates in one subgroup and the other four Slovenian isolates in another. Nonetheless, both Montenegrin isolates are clustered again into a distinct subgroup with similar Italian and Asian group of accessions as for the GcGn gene. The average nucleotide sequence identity is similar for NSm (98.2%) as for GcGn gene within the clade M3 and the range of average nucleotide sequence identities is 93.4–96.0% between the three clades (Supplementary Table S5).

Despite some incongruence, the overall phylogenetic tree topologies for the N and NSs genes in the S segment again reflect the clade S3 grouping (Siampour et al., 2024). Slovenian and Croatian isolates are grouped into the same subgroup, while two Montenegrin isolates are more distant and grouped with a geographically distant accession—the Chinese YNHHMZ isolate from tobacco (Supplementary Table S3; Figure 4). The isolates from Croatia and Slovenia are again grouped closely together making their own respective subgroups for the NSs and N gene with the addition of a small number of mostly Italian isolates. The average nucleotide sequence identities within the clade S3 for N and NSs genes are 98.8 and 98.4%, respectively (Supplementary Table S5). The average nucleotide sequence identities within the clade S3 for N and NSs genes are 98.8 and 98.4%, respectively (Supplementary Table S5). Intergroup variability between cluster range 96.5–97.0% for N gene, and 94.9–95.9% for NSs gene (Supplementary Table S5).

**Figure 5:**
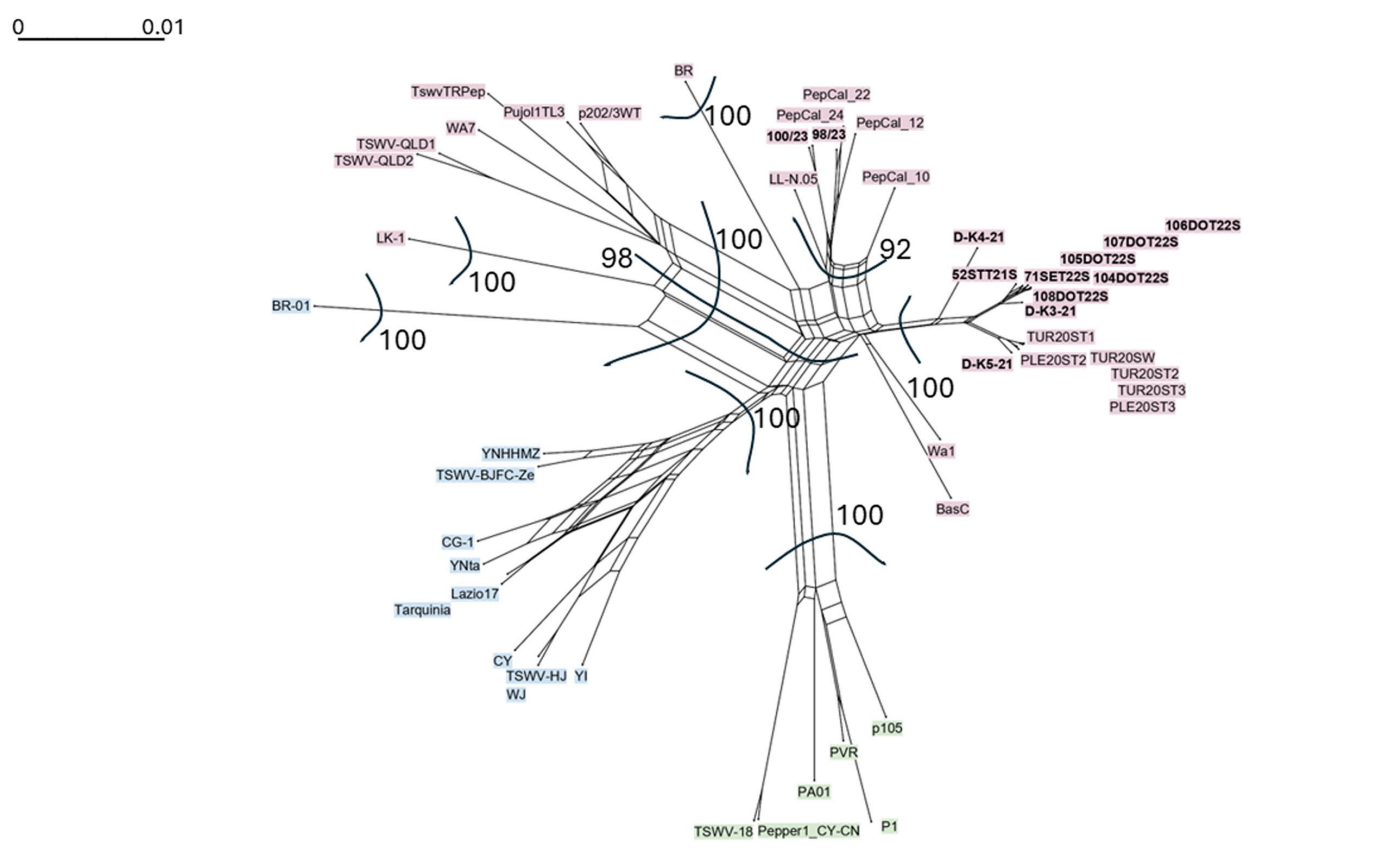
Phylogenetic network of concatenated gene sequences of TSWV isolates. The networks were created with SplitsTree 5 using the neighbor-net algorithm. The scale bar shows how the length of a branch translates in sequence divergence. The scale bar indicates the number of nt substitutions per site. The TSWV isolates generated in this study are in bold. Isolates of clade L1 are shown in pink, isolates of clade L2 in blue and isolates of clade L3 in green. Bootstrap support is only given for splits separating main groups.

The phylogenetic network analysis of concatenated sequences of all five genes (RdRp, GcGn, NSm, N and NSs) of three TSWV Slovenian, seven Croatian and two Montenegrin isolates (Supplementary Table S4) shows reticulation among clades of TSWV. The analyses revealed well separated groups (Figure 5). Isolates obtained in this study from Croatia and Slovenia grouped together, with Slovenian isolates from previous study (Rivarez et al., 2023), while Montenegrin isolates grouped with four Italian isolates and one isolate from Spain. The network displayed a reticulate topology indicating possible recombination or reassortment events among the TSWV isolates. Reticulation patterns confirm the observations from phylogenetic analyses of separate segments and indicate that isolates from this study are potentially reassortants between isolates from Clade 3 and a group of isolates from Clade 1 (from United States, Turkey, South Africa, and Australia) (Figure 5).

### Recombination analysis

Recombination analysis using RDP4 software supported the findings from phylogenetic and network analyses. Among the 12 isolates with complete gene sequences, 11 showed evidence of reassortment events, while one isolate (D-K4-21) was identified as a potential major parent. These results suggest that reassortment, rather than true recombination, has played a role in shaping the genetic diversity of TSWV isolates in this study (details in Supplementary Tables S6, S7).

## Discussion

Tomato cultivation in Croatia, Slovenia and Montenegro is an important aspect of vegetable production. According to the available data, tomato production in 2023 in Croatia was taking up 440 ha, in Slovenia 220 ha, and in Montenegro 48 ha (FAOSTAT, 2025). Economically, TSWV is also the most devastating virus of tomato and peppers in the Mediterranean basin (Turina et al., 2012) and has been reported as a concern in all three countries in tomato and other crops (Mavrič and Ravnikar, 2001; Zindovic et al., 2014; Ivić et al., 2016; Rivarez et al., 2023). Apart from preliminary data (Škorić et al., 2023) and available uncharacterised sequence data for several Slovenian TSWV isolates (Rivarez et al., 2023), there has been no systematic effort to investigate complete TSWV genomic data from infected tomato across this three-state area. This joint study aimed to analyze and compare the molecular diversity of TSWV isolates occurring in tomato across Slovenia, Croatia, and Montenegro considering samples from the last five vegetative seasons.

Our sampling included continental and coastal climatic zones, tomatoes from open fields and protected facilities, and distinct cultivars. They had different levels of TSWV resistance, as declared by the seed producers (Supplementary Table S1) but all had TSWV infection-like symptoms. Screening procedures resulted in 18 libraries of pooled and single samples (Supplementary Figure S1) for HTS. Analysis of 79 TSWV genomic segment sequences (Figures 2–4) was performed reflecting this distribution by country. Complete genome sequences of TSWV were reconstructed for seven Croatian, three Slovenian and two Montenegrin RdRp-GnGc-NSm-N-NSs gene concatenated sequences. Nevertheless, the phylogenetic relationships of isolates across the three countries are consistent for separate genomic segments (Figures 2–4) as well as for a limited-number of whole genomes in the phylogenetic network analysis (Figure 5). The RdRp gene analysis (Figure 2) shows the clustering of TSWV isolates studied here with most of the European isolates used in the study, belonging to clade L1 or D-like (Fontana et al., 2020) group. The clustering of two Montenegrin full genome and RdRp sequences within the clade L1 subgroup, alongside predominantly Mediterranean isolates (from Italy and Spain) and distinct from Slovenian and Croatian isolates, is a pattern that is also observed in the analyses of the other two genomic segments.

Unlike the RdRp gene analysis, the phylogenetic analysis of the M and S segment genes (Figures 3, 4) positioned our isolates within clades M3 and S3, respectively (A-like, Fontana et al., 2020). The average within and between nucleotide identity matrices (Supplementary Table S5) also demonstrate these relationships. Divergent subgrouping of the two Montenegrin isolates, or the origin of remaining isolates do not affect their main grouping into the same clade. However, it suggests that the origin of these isolates may be a different introduction compared to the origin of Croatian and Slovenian isolates.

All tomato TSWV genotypes analyzed here can be described as L1-M3-S3 according to a very recent global TSWV genotyping study (Siampour et al., 2024). Most European TSWV isolates analyzed in that study had L1-M1-S1, L3-M1-S1, and L1-M3-S3 genotypes probably resulting from reassortment events in their evolutionary past. Interestingly, the subset of genotypes originating from cultivated solanaceous plants, but mostly from peppers, had L1-M3-S3 genotype (Siampour et al., 2024). Typing of all tomato TSWV sequences in this study as members of L1-M3-S3 genotype confirms this link to solanaceous vegetables. We suppose that the same predominant reassortant TSWV genotype has been circulating over the last 5 years in this three-country Euro-Mediterranean zone. It would be interesting to explore the diversity and evolutionary forces shaping populations of TSWV in this zone and whether the L1-M3-S3 tomato TSWV genotype here results from adaptation to tomato. This is underlined by the finding of a genotype (109DOP22S, Škorić et al., unpublished) denoted as L3-M1-S1 by Siampour et al. (2024) in peppers cultivated side by side to the tomato PE tunnel in Domašinec, and of note sampled at the same time as tomatoes (Supplementary Table S1).

The SplitsTree phylogenetic network analysis (Figure 5) confirmed the results of the phylogenetic trees, showing the grouping of the isolates generated in this study in two close groups. Montenegrin isolates together with Italian and Spanish isolates, and Croatian and Slovenian isolates together, with other Slovenian isolates from a recent study by Rivarez et al. (2023). A reticulated phylogenetic network generated from concatenated sequences points to conflicting phylogenetic signals within the alignment which could suggest that TSWV evolutionary history was shaped by recombination and reassortments (Huson and Bryant, 2005). Taking into account the results of the phylogenetic studies, which show different classification of L segment (RdRp gene), M and S segment genes (L1 vs. M3 and S3), reassortment probably plays a more significant role in the evolution of isolates from this study, as suggested for TSWV previously in the literature (Tentchev et al., 2011; Lian et al., 2013; Fontana et al., 2020; Siampour et al., 2024). The analysis of potential recombination events with RDP4 software (Supplementary Tables S6, S7) revealed evidence of recombination breakpoints in L-segment of 11 isolates from this study, based on the concatenated (RdRp-GnGc-NSm-N-NSs) sequences. Recombination was detected by seven out of the nine programs used in RDP4 (Martin et al., 2015) and confirmed the results of phylogeny and network analyses.

When the phylogenetic relationships of the sequences of isolates circulating in each of these countries are further examined, all Croatian gene sequences cluster closely together in the analyses of all genes (Figures 2–4). This could be expected for most of the Croatian tomato samples because they were taken from the same greenhouse in Domašinec (northwestern Croatia) in 2022 (Supplementary Table S1) during a severe disease outbreak. Interestingly, the genotype of the sample 52STT21S from central Dalmatia (sampled in 2021), which is geographically and temporally distant, as well as the geographically distant isolate 71SET22S from Eastern Croatia (Supplementary Table S1), consistently cluster closely with sequences originating from the greenhouse in Domašinec. This could be interpreted as a uniform tomato population of TSWV circulating in the country in recent years. Also, as main thrips vectors could hardly cross the mountain barrier separating coastal (Dalmatia) and continental localities on their own, one could speculate earlier exchange of infected tomato, or ornamental, planting material and establishment of the viral populations locally (Kirk and Terry, 2003; Szostek et al., 2017). More research and archival samples are likely needed to test this hypothesis. However, Slovenian samples were sourced over the greater time span, and they do not diverge much from each other or the ones from neighboring Croatia (Supplementary Table S1; Figures 2–4).

Unlike the highly similar populations circulating in tomatoes across different geographically well-separated agro-ecological niches and vegetative seasons in Croatia and Slovenia, the two Montenegrin isolates consistently branch more distantly within the same phylogroups. It is evident that these two isolates are divergent from Croatian-Slovenian group. They are positioned closer to Asian non-tomato (e.g., Chinese tobacco isolate YNHHMZ) or some Italian and Spanish pepper isolates, depending on the analyzed gene sequence (Figures 2–4). However, there are only two Montenegrin accessions in this study. Such a small sample set undoubtedly introduces bias and precludes drawing conclusions on the TSWV diversity in the whole country. Even so, several questions may be entertained here. One of them being: was the introduction source of the two Montenegrin TSWV isolates separate and different than for Croatian and Slovenian isolates or was it just derived by chance or selection as a divergent subgroup from a common starting TSWV population? As Chinese sequences are the most closely related to Montenegrin subset of samples in some of the analyses, could China be their country of origin? The “Chinese” TSWV gene flow hypothesis has been already proposed for some of the Italian pepper TSWV isolates (Fontana et al., 2020). It is not so far-fetched given that many seed companies have their production in Chinese subsidiaries, including the producer of Pink Gusto F1 cultivar sampled in Montenegro.^4^ While TSWV seed transmission is not likely (EPPO, 2025), the import and establishment of other vegetable and ornamental commodities could have facilitated TSWV genetic exchange (Wang et al., 2022). Lastly, more extensive TSWV genetic analysis in this country is necessary to obtain the balanced number of samples over longer time, possibly including samples collected before 2023, to answer some of these questions.

Regardless of severe symptoms (Supplementary Figure S1) observed in many tomatoes sampled for this study, even from cultivars with declared intermediate or high TSWV resistance (Supplementary Table S1), we found no evidence of mutations in the NSm gene (Figure 1) resulting in aa substitutions described for RB TSWV strains so far (Huang et al., 2022; Almási et al., 2023; Rodríguez-Negrete et al., 2023). The same observation was reported for TSWV strains from tomato in Texas, capable of disrupting the *Sw-5* mediated resistance however not carrying the classical substitutions in the NSm protein (Chinnaiah et al., 2023), therefore opening hypothesis on putative novel, unknown mechanisms involved in resistance breaking. Even though the type and severity of viral symptoms are important for the economic impact of TSWV, they do not result only from viral genotype but from complex plant-virome-environment interactions (García-Cano et al., 2006; Alcaide et al., 2021). These interactions were not investigated *per se* in this study, however the presence of other viral reads in metagenomic data (Supplementary Table S1) suggest the possibility that other virome constituents (e.g., PVY, CMV, etc.) contributed to the development of the observed symptoms.

In conclusion, our study reveals that isolates of TSWV infecting tomatoes in Croatia, Montenegro, and Slovenia have genotypes comparable to the most European isolates, described as L1-M3-S3, common for solanaceous vegetables produced in Europe. In addition, the two Montenegrin isolates are genetically distinct from Slovenian and Croatian isolates, suggesting a separate introduction event. Despite sometimes severe disease symptoms observed on sampled tomatoes, no evidence of RB strains has been found based on known molecular markers in the NSm protein. Understanding the genetic diversity of TSWV is crucial for its host adaptation, vector transmissibility, and other molecular and biological characteristics significant in epidemiology. The molecular data on TSWV circulating across the three countries provides an overview of the TSWV genetic pool, whose understanding is essential for informing breeding programs and formulating sustainable management strategies, through support in the identification of tomato genotypes of interest to counteract TSWV and mitigate occurrence of future outbreaks.

## Data Availability

The raw RNA sequencing data were submitted to the National Center for Biotechnology Information (NCBI) SRA database under accession numbers PRJNA946736 and PRJNA1219120 and the 58 genomic sequences of individual isolates are freely available in the NCBI GenBank under the accession numbers listed in the Table 1.

## Footnotes

^1^ https://ictv.global/taxonomy

^2^ https://www.ebi.ac.uk/

^3^ https://itol.embl.de

^4^ https://yukselseeds.com/en/about-us
