# Correction: Tomato spotted wilt virus in tomato from Croatia, Montenegro and Slovenia: genetic diversity and evolution

**DOI:** 10.3389/fmicb.2026.1901047

**Published:** 2026-07-10

**Authors:** Dijana Škorić, Jelena Zindović, Dorotea Grbin, Patrik Pul, Vladan Božović, Paolo Margaria, Nataša Mehle, Anja Pecman, Zala Kogej Zwitter, Denis Kutnjak, Ana Vučurović

**Affiliations:** 1 Department of Biology, Faculty of Science, University of Zagreb, Zagreb, Croatia; 2 Department of Plant Protection, Biotechnical Faculty, University of Montenegro, Podgorica, Montenegro; 3 Faculty of Food Technology, Food Safety and Ecology, University of Donja Gorica, Podgorica, Montenegro; 4 Plant Virus Department, Leibniz Institute DSMZ GmbH, Braunschweig, Germany; 5 Department of Biotechnology and Systems Biology, National Institute of Biology, Ljubljana, Slovenia; 6 School of Viticulture and Enology, University of Nova Gorica, Nova Gorica, Slovenia; 7 JoŽef Stefan International Postgraduate School, Ljubljana, Slovenia

**Keywords:** tomato, HTS, phylogeny, TSWV, plant virus

The funder Slovenian Research and Innovation Agency (ARIS) Slovenian Croatian Bilateral Project, BI-HR/23-24-038 was erroneously omitted.

The corrected **Funding** Statement is below:

“The author(s) declare that financial support was received for the research and/or publication of this article. This research was funded by the Croatian Science Foundation grant NanoPhyto (HRZZ IPS-2020-01-2960), the Slovenian Research and Innovation Agency (ARIS), Republic of Slovenia Core Financing (P4-0165 and P4-0407), Slovenian Montenegrin Bilateral Project (BI-ME/23-24-028), Slovenian Croatian Bilateral Project (BI-HR/23-24-038), the Slovenian Ministry of Agriculture, Forestry, and Food through the Administration of the Republic of Slovenia for Food Safety, Veterinary Sector and Plant Protection, the European Virus Archive Global (EVAg) project through the European Union's Horizon 2020 research and innovation program under grant agreement number 871029, and Euphresco project 2020-A-343.”

The original version of this article has been updated.

