# Supplementary material for: Tomato spotted wilt virus in tomato from Croatia, Montenegro and Slovenia: genetic diversity and evolution
Source: Front Microbiol. 2025 Jul 28;16:1618327. doi: 10.3389/fmicb.2025.1618327 (PMC12336143; doi:10.3389/fmicb.2025.1618327)
Supplement: Supplementary file 1 [file Data_Sheet_1.docx]

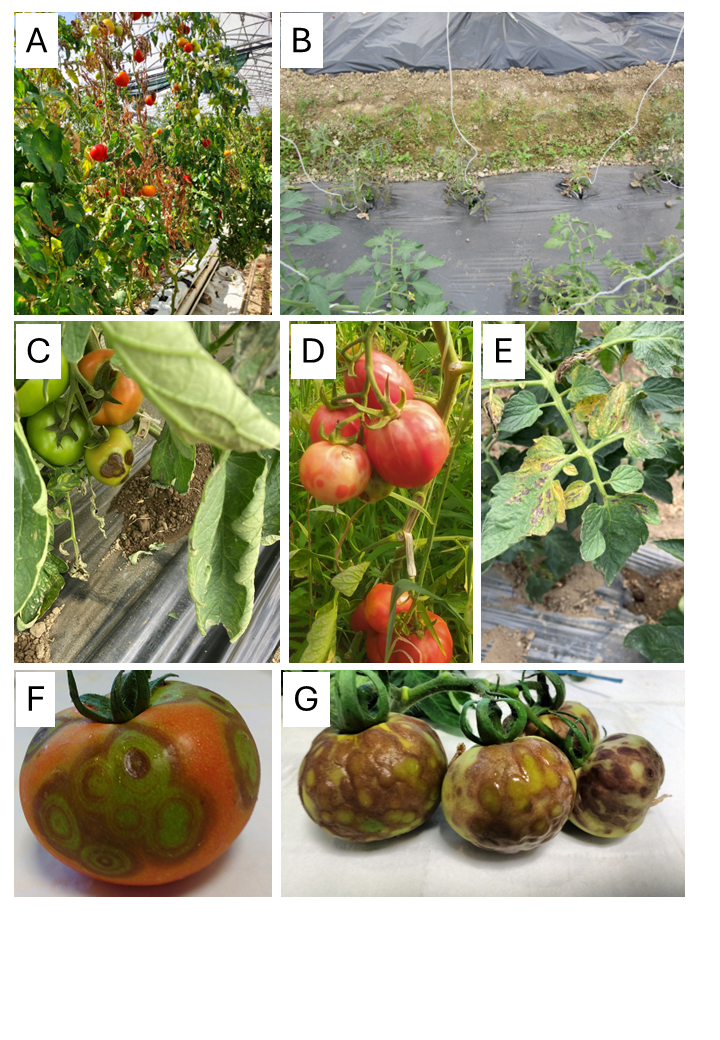


Supplementary figure 1. Possible disease symptoms on the leaves and fruits of sampled tomato plants. **A,** Plant wilting (52STT21S, Photo: Adrijana Novak, HAPIH, Croatia). **B,** Stunted growth (D262-20, Photo: Magda Rak-Cizej, IHPS, Slovenia). **C,** Wilting of leaves and necrotic ring spots on fruits (105DOT22S, Photo: Dijana Škorić, Croatia). **D,** Fruit marbling and uneven ripening (98/23, Photo: Sandra Džoganović, Montenegro). **E,** Chlorotic and necrotic patches on leaves (105DOT22S, Photo: Dijana Škorić, Croatia). **F,** Ringspots on fruits (D599/20, Photo: Nejc Jakoš, NIB, Slovenia). **G,** Severe necrosis of fruits (D599/20, Photo: Nejc Jakoš, NIB, Slovenia).
