# Supplementary material for: Tomato spotted wilt virus in tomato from Croatia, Montenegro and Slovenia: genetic diversity and evolution
Source: Front Microbiol. 2025 Jul 28;16:1618327. doi: 10.3389/fmicb.2025.1618327 (PMC12336143; doi:10.3389/fmicb.2025.1618327)
Supplement: Supplementary file 3 [file Table_2.docx]

**Supplementary Table 2**. The Number of trimmed reads, average length of trimmed reads and the length of the most abundant sRNA class for the samples sequenced in this study

| **Designation of the library** | **Sequencing approach** | **Raw reads count** | **Average reads length before trimming** | **Reads count after trimming and filtering** | **Average reads length after trimming** |
| --- | --- | --- | --- | --- | --- |
| D-K3-21 | Illumina | 32078928 | 150.0 | 31981154 | 149.63 |
| D-K4-21 | Illumina | 34244168 | 150.0 | 34146398 | 149.67 |
| D-K5-21 | Illumina | 36360298 | 150.0 | 36258759 | 149.66 |
| D-P4-22 | ONT | 2009049 | 236.5 | 888281 | 262.8 |
| D-P10-22 | ONT | 782456 | 209.4 | 221680 | 286 |
| 98/23 | Illumina | 24968990 | 146 | 23261710 | 137.1 |
| 100/23 | Illumina | 25163028 | 147 | 23318268 | 137.3 |
| D262/20 | Illumina | 2563442 | 251.0 | 2563423 | 235.5 |
| D474/20 | Illumina | 2378970 | 251.0 | 2378950 | 233.7 |
| D599/20 | Illumina | 2618958 | 251.0 | 2618944 | 231.5 |
| D-P2-24 | ONT | 1909204 | 466.1 | 1892858 | 399.5 |
| 52STT21S | Illumina | 12420584 | 150.0 | 12280065 | 147.94 |
| 104DOT22S | Illumina | 11400529 | 150.0 | 11400423 | 148.1 |
| 105DOT22S | Illumina | 8218820 | 150.0 | 8217650 | 135 |
| 106DOT22S | Illumina | 8273817 | 150.0 | 81272197 | 135 |
| 107DOT22S | Illumina | 10968607 | 150.0 | 10934562 | 135 |
| 108DOT22S | Illumina | 16659127 | 150.0 | 16659015 | 149.5 |
| 71SET22S | Illumina | 14312351 | 150.0 | 14311121 | 142.6 |
