# Supplementary material for: Tomato spotted wilt virus in tomato from Croatia, Montenegro and Slovenia: genetic diversity and evolution
Source: Front Microbiol. 2025 Jul 28;16:1618327. doi: 10.3389/fmicb.2025.1618327 (PMC12336143; doi:10.3389/fmicb.2025.1618327)
Supplement: Supplementary file 6 [file Table_5.docx]

Supplementary Table 5. The average nucleotide sequence identities of the RdRp, GcGn, NSm, N and NSs genes within (table cells shaded in grey) and between different phylogenetic groups of TSWV.

| **Segment** | **Gene** | **Phylogroup** | **Clade L/M/S 1** | **Clade L/M/S 2** | **Clade L/M/S 3** |
| --- | --- | --- | --- | --- | --- |
| **L** | **RdRp** | **Clade L1** | 98.0 |  |  |
|  |  | **Clade L2** | 93.9 | 97.7 |  |
|  |  | **Clade L3** | 94.0 | 95.6 | 98.1 |
| **M** | **GcGn** | **Clade M1** | 97.2 |  |  |
|  |  | **Clade M2** | 93.7 | 98.7 |  |
|  |  | **Clade M3** | 93.4 | 96.4 | 98.0 |
|  | **NSm** | **Clade M1** | 97.2 |  |  |
|  |  | **Clade M2** | 93.4 | 98.9 |  |
|  |  | **Clade M3** | 93.6 | 96.0 | 98.2 |
| **S** | **N** | **Clade S1** | 98.4 |  |  |
|  |  | **Clade S2** | 96.7 | 97.9 |  |
|  |  | **Clade S3** | 97.0 | 96.5 | 98.8 |
|  | **NSs** | **Clade S1** | 97.9 |  |  |
|  |  | **Clade S2** | 95.9 | 97.7 |  |
|  |  | **Clade S3** | 95.0 | 94.9 | 98.4 |
