# Supplementary material for: Tomato spotted wilt virus in tomato from Croatia, Montenegro and Slovenia: genetic diversity and evolution
Source: Front Microbiol. 2025 Jul 28;16:1618327. doi: 10.3389/fmicb.2025.1618327 (PMC12336143; doi:10.3389/fmicb.2025.1618327)
Supplement: Supplementary file 7 [file Table_6.docx]

Supplementary table 6. Summary of recombination analysis of concatenated TSWV isolates obtained in this study, along with representative GenBank accessions. The table includes predicted recombinant sequences, recombined genomic segments, parental sequences, and statistical support from multiple detection methods implemented in the RDP software.

| **Recombinant Sequence(s)** | | PepCal_10, **52STT21S,** TUR20SW, TUR20ST2, TUR20ST1, TUR20ST3, PLE20ST3, PLE20ST2, **D-K3-21, D-K5-21, 71SET22S,** **105DOT22S, 104DOT22S, 107DOT22S, 108DOT22S**, **106DOT22S, 98/23, 100/23,** PepCal_22 |
| --- | --- | --- |
| **Recombined segment** | | L |
| **Breakpoints** | **Begin** | 54 |
|  | **End** | 8604 |
| **Minor Parent** | | Pujol1TL3, TSWV-QLD2, TSWV-QLD1, WA7, BR, PepCal_12, p202/3WT, PepCal_24 |
| **Major Parent** | | p105, **D-K4-21** |
| **RDP** | | 7.59E-10 |
| **GENECONV** | | 3.77E-09 |
| **Bootscan** | | 1.24E-09 |
| **Maxchi** | | 2.34E-11 |
| **Chimaera** | | 2.54E-09 |
| **SiSscan** | | 5.99E-54 |
| **PhylPro** | | NS |
| **LARD** | | NS |
| **3Seq** | | 1.44E-61 |

*NS - No significant P-value was recorded for this recombination event using this method.
